# Condylar intramedullary intraosseous lipoma: Contribution of a new case and review of the literature

**DOI:** 10.4317/jced.53421

**Published:** 2017-03-01

**Authors:** Alba Sanjuan, Alicia Dean, Blas Garcia, Francisco Alamillos, Elisa Roldan, Antonio Blanco

**Affiliations:** 1 MD, Oral and Maxillofacial Surgery Department, Reina Sofía University Hospital. Maimonides Biomedical Research Institute. Cordoba. Spain; 2 MD, Head, Oral and Maxillofacial Surgery Department, Reina Sofía University Hospital. Maimonides Biomedical Research Institute. University of Cordoba, School of Medicine. Cordoba. Spain; 3 MD, Assistant, Oral and Maxillofacial Surgery Department, Reina Sofía University Hospital. Maimonides Biomedical Research Institute. University of Cordoba, School of Medicine. Cordoba. Spain; 4 MD, Assistant, Radiology Department, Reina Sofía University Hospital. Maimonides Biomedical Research Institute. Cordoba. Spain; 5 MD, Dentist, Health district, Córdoba - Guadalquivir, Andalucian Health Service. Maimonides Biomedical Research Institute. University of Cordoba. Cordoba. Spain

## Abstract

**Background:**

Lipoma is the most common benign tumour of the human body, being intraosseous involvement very rare. Just 1 to 4% of all cases of lipoma are located in the oral cavity, only 0.1% being intraosseous. The jaw is its most uncommon bone location. Etiology of intraosseous lipoma (IOL) is unknown, although several theories have been proposed. Usually asymptomatic, the symptoms, when present, will depend on its location and size. Its origin may be intraosseous or juxtacortical. A biopsy is essential for diagnosis, and definitive treatment involves resection or curettage of the lesion. The aim of this paper is to present a new case of intramedullary intraosseous lipoma of the mandible with involvement of the left mandibular ramus and condylar neck.

**Material and Methods:**

A case of intramedullary intraosseous lipoma (IOL) on the left mandibular ramus and condyle is presented. No history of trauma in temporomandibular joint existed. The radiology showed a radiolucent multi-lobulated lesion with values of attenuation in the range of fat. Curettage is performed and the histopathology showed a conglomerate of adipocytes without trabeculae, calcifications or atypia.

**Results:**

According to the bibliography 24 cases of mandibular IOL have been described. This is the second reported case of condylar involvement and the first with cortical expansion.

**Conclusions:**

Lipoma intraosseous is a very rare benign bone neoplasm. Histology is required for the differential diagnosis from other radiolucent lesions. The IOL treatment is the curettage with a good prognosis, although malignant transformation to liposarcoma has been reported in other locations. It is a disease with a difficult differential diagnosis, therefore the publication of new cases is important.

** Key words:**Intraosseous lipoma, lipoma, jaw tumour, condylar tumour.

## Introduction

Lipoma is a circumscribed, slowly growing benign mesenchymal tumour, formed by a conglomerate of mature adipocytes without cell atypia. It can be found in multiple locations due to the wide distribution of fat tissue throughout the body. Subcutaneous lipoma is the most frequent clinical presentation, but lipoma may be also found at intramuscular, retroperitoneal and intraosseous levels (1). Oral cavity affectation just represents 1 to 4.5% of all benign oral cavity tumours. Lipoma is one of the less common bone tumours, accounting for 0.1% of them (1,2). Within the oral cavity, they have been reported to occur in the buccal mucosa (45.7%), tongue (13%), lips (13%) and floor of the mouth (10.9%), among other areas (3,4). The most common location of intraosseous lipoma is the medullary bone of the calcaneus and the metaphysis of long bones, the jaw being considered an exceptional location (5).

The first case of mandibular intraosseous lipoma (IOL) was reported by Oringer in 1948 as a radiolucent lesion under a second molar; when chewing or exerting pressure in the molar region pain was elicited (6,7). The most frequent location within the jaw is the tooth bearing area, appearing in the symphysis, the body and the ramus. Maxillay involvement has also been reported (8-10). Mandibular lipoma tends to affect those between the fourth and the sixth decade of life. It is more common in males than females with a ratio of 1.6:1 (6,11). Most cases are asymptomatic, being diagnosed by chance during a radiographic examination. Symptoms depend on its size, location, time of evolution and growth rate. Pain, swelling, and numbness may occur. They usually appear as a uni- or multi-locular radiolucent lytic lesion, and thus a differential diagnosis with other benign and malignant lytic lesions should be made (6,12,13). Etiology of IOL is not clear; some authors have proposed that it may be related to osteoporotic bone or ischemic trauma, and others consider it to be a new onset as benign neoplasm. Treatment involves curettage of the lesion, with or without grafting the cavity (1,2). IOLs are classified as intramedullary if they arrive from the fat of intramedullary bone, intracortical if they have their origin in the cortical bone and juxtacortical if they originate in the periosteum, or the soft tissue surrounding the bone (1,4,5,8,9). The aim of this paper is to report a new case of mandibular IOL along with a review of the literature and an update of the pathogenesis, symptoms, radiologic images, management and current treatment.

## Case Report

**Figure 1:**
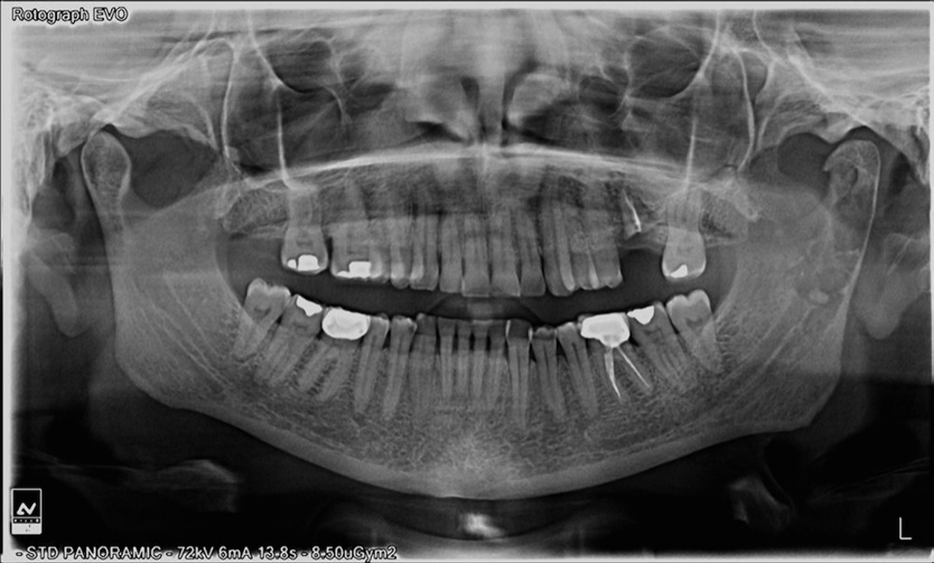
Ortopantomography. Left mandibular ramus and left condylar neck radiolucid lesion.

**Figure 2:**
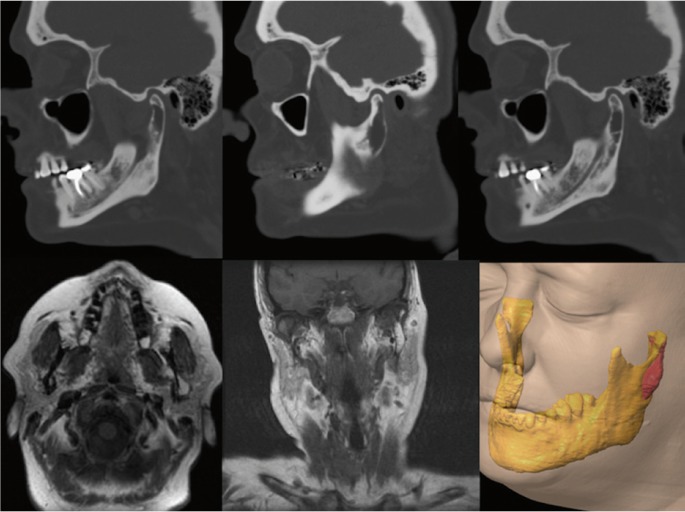
Sequence of CT and MRI images with expansive lytic lesion in which cortical reabsorption can be seen. Hyperintense images in T1 and T2 sequences, corresponding to fat. Image of the Lesion in 3D acquired by the software iPlan 3.0 ( BrainLab).

**Figure 3:**
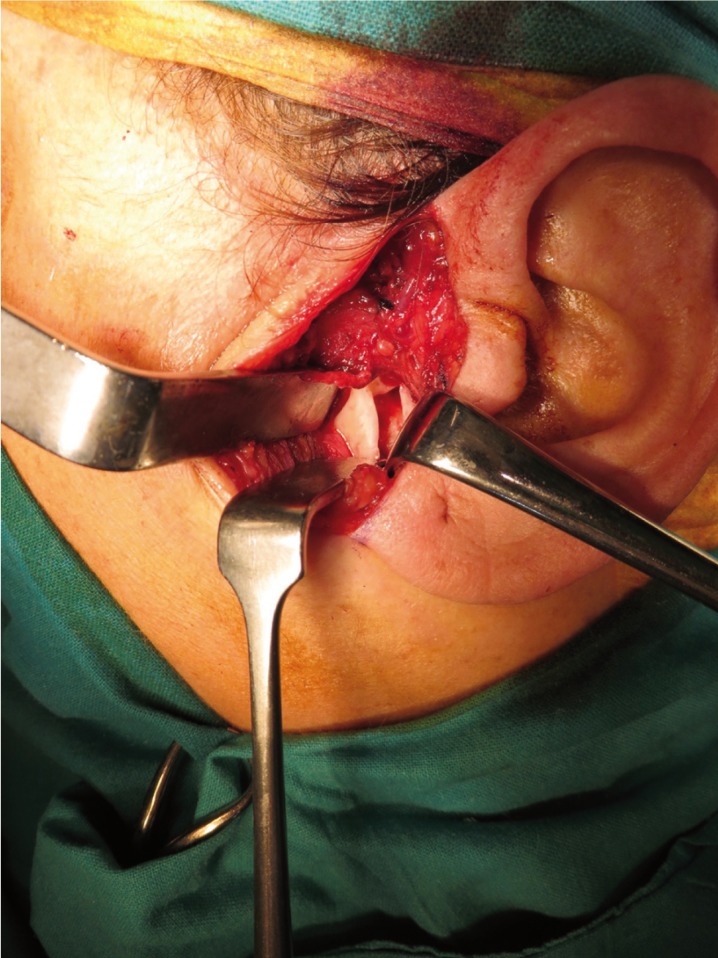
Intraoperative view of the lesion through cortical neck bone reabsorption.

A 50 year-old female was referred to our service because of a radiolucent image on the left mandibular ramus and condyle discovered in a radiographic examination. No history of temporomandibular joint trauma or pathology was present. An orthopantomography showed a radiolucent, multi-lobed, well circumscribed lesion, in the left mandibular ramus and condyle (Fig. 1). A computed tomography scan (CT) showed a well-defined multi-lobed radiolucent lesion with septa within, of 41 x 11mm craniocaudal and anteroposterior axes respectively, with values of attenuation in the range of fat. The lesion was located posterior to the alveolar nerve channel, and a thinning and a focal discontinuity of the vestibular cortex could be appreciated in the mandibular ramus. A magnetic resonance imaging (MRI) revealed a hyperintense lesion in T1 and T2 weighted sequences, a typical signal of fat (Fig. 2). All these features were consistent with the diagnosis of intraosseous lipoma. A biopsy under general anaesthesia using a preauricular approach was performed. During surgery, a well-defined resorption of the external cortex of the left mandibular ramus and the condylar neck could be seen, as had been anticipated by the CT image. The lesion was located in the mandibular medullar cavity and had the appearance of fatty tissue. Resection was performed by curettage and filling the defect was not considered to be necessary. The histopathology showed a conglomerate of adipocytes without bone trabeculae, calcifications or atypia (Fig. 3). The pathological findings confirmed the definitive diagnosis of intraosseous lipoma. Now, after 20 months, the patient remains free of symptoms and shows no sign of recurrence. Patient inform consent has been obtained for the publication of this article.

## Discussion

Mandibular IOL is very rare, with only twenty-three cases reported in the literature ( Table 1). Only one other case of IOL affecting the mandibular condyle has been reported, in which there is no expansion or cortical involvement, unlike the case that we show (9).

**Table 1:**
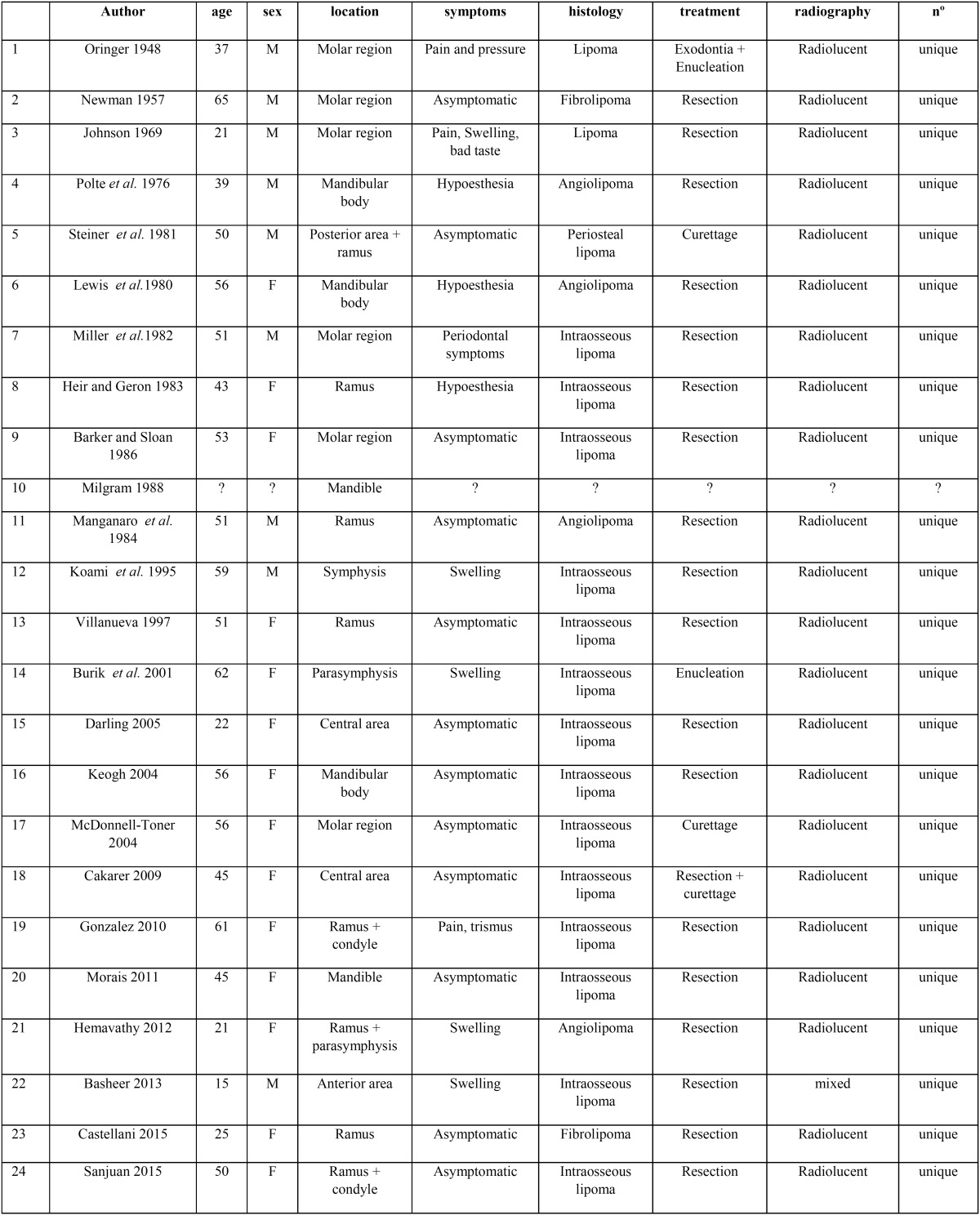
Summary of location, clinical features, x-ray findings and treatment of the published cases of Intraosseous lipoma of the jaw since 1948.

In 1969, Hart studied the location of intraosseous lipoma and its relationship to the bone, establishing four categories: lipoma from the surrounding soft tissue, intraosseous lipoma of the intramedullary cavity, periosteal lipoma which may deform the bone by pressure or periosteal reaction, and the liposarcoma, a malignant tumour with local destruction and possible distant metastasis (11). In 2001, Burik classified mandibular IOL according to its 3 possible origins: medullar cavity fat (intramedullary lipoma), periosteum (periosteal lipoma) and, less likely, the adjacent soft tissue that could invade the bone secondarily and appears as a periosteal lipoma (6). Basheer (2013) differentiates between true intraosseous lipomas, from medullary or cortical fat bone (intramedullary or intracortical) and juxtacortical lipomas from soft tissue or periosteum (1).

Etiology of IOL remains unclear; dental trauma, disruption of the post-extraction healing process, retention of radicular remains (9), medullary bone infarction (common in elderly) or osteoporotic bones have been considered as possible etiological factors (1,4). Most IOLs are asymptomatic; but they can cause pain, swelling or numbness, depending on their growth rate and their relationship to other structures such as mandibular tooth roots, the dental nerve, etc (1,2,4,6,8).

The intramedullary mandibular IOL shows up on x-rays as a well-circumscribed radiolucent uni- or multi-locular lesion, with possible central calcification or ossification and a partial or complete sclerotic border, which may or may not be associated with cortical expansion. A CT scan shows a homogeneous density equal to the density of fat, although heterogeneity may also appear when myxoid degeneration or calcification develops. Marginal sclerosis and cortical irregularity can also be seen, although re-sorption is a rarity. In our case, a lytic image producing a thinning of cortical bone and a focal disruption of the vestibular cortex in the mandiblar ramus was seen. On MRI a similar homogeneous signal intensity with fat density was seen in both T1 and T2. No cases of multiple bone lesions have been reported (5).

The histopathology is characterized by presence of mature adipocytes without atypia and hematopoietic tissue, which may be encapsulated. Milgram established three stages based on the viability of the fatty tissue and the presence of calcification or bone trabeculae: stage 1 presents absence of them, stage 2 presents partial necrosis and calcification, with viable adipose tissue remaining, and stage 3 presents complete necrosis and tissue involution, with dystrophic calcification, and a greater predisposition to malignancy (14,15). Our case belongs to stage 1 (6,9,14). Several histopathologic varieties have been reported: simple lipomas, fibrolipomas, angiolipomas, pleomorphic, myxoid lipomas and spindle cell lipomas. In the mandible fibrolipomas and intraosseous angiolipomas (2,4,14) have also been described.

The differential diagnosis must be performed with other radiolucent bone images; such as the simple cyst, post-traumatic cyst, aneurysmal bone cyst, giant cell granuloma, ameloblastoma, osteoblastoma, arteriovenous malformations, hemangiomas, infarcted bone, chondrosarcoma or liposarcoma (2-4).

The treatment consists of curettage with or without bone graft or filling, which in our case was not necessary. Other techniques have been used such as phenolization, without much success with regard to the surgery. Only four cases of malignancy localized in the tibia, fibula and femur have been reported, but no case of mandibular IOL malignant transformation or recurrence (15). In conclusion, IOL is a very rare benign bone neoplasm. It is usually asymptomatic. Histology is required for the differential diagno-sis from other radiolucent lesions. Treatment consists of curettage and subsequent bone filling when necessary. The prognosis is very good, although malignant transformation to liposarcoma has been reported in other locations. It is a disease with a difficult differential diagnosis, therefore the publication of new cases is important.
